# Reperfusion Therapies in Acute Ischemic Stroke Beyond the Conventional Time Window: A Narrative Review

**DOI:** 10.7759/cureus.45864

**Published:** 2023-09-24

**Authors:** Mohammad Mansour

**Affiliations:** 1 Department of General Medicine, University of Debrecen, Debrecen, HUN; 2 Department of General Medicine, Jordan University Hospital, Amman, JOR

**Keywords:** flair, perfusion ct, diffusion-weighted imaging, acute ischemic stroke, mechanical thrombectomy, intravenous thrombolysis, penumbra, stroke, reperfusion therapies

## Abstract

Stroke is the second most common cause of death worldwide, with 50% of survivors experiencing long-term disability. For more than two decades, treatment with intravenous thrombolysis (IVT) and mechanical endovascular thrombectomy (MET), the only approved stroke reperfusion therapies, was restricted to patients within the 4.5-6 hour time window, respectively. Therefore, patients who presented with acute ischemic stroke (AIS) beyond the conventional time window were excluded from reperfusion treatment. This narrative review aims to review the scientific literature on the possibilities of reperfusion therapies for patients who present with an unknown time of stroke onset, and those with stroke onset beyond the conventional 4.5-6 hour time window. Beyond the conventional time window, the eligibility of patients for IVT or MET, the two main therapeutic procedures, is decided based on the concept of penumbral imaging. Penumbral imaging identifies patients with hypoperfused but viable brain tissue, who could benefit from reperfusion. On the other hand, clock-based DWI-fluid-attenuated inversion recovery (FLAIR) magnetic resonance imaging (MRI) can detect stroke that has occurred within 4.5 hours in patients with an unknown time of onset, including patients who awaken with stroke. The introduction of penumbral imaging and MRI-based tissue clocking as imaging biomarkers for stroke has revolutionized stroke therapy, potentially allowing for personalized treatment of eligible stroke patients.

## Introduction and background

Stroke is an umbrella term that refers to conditions in which damage to the brain tissue or spinal cord occurs due to a vascular origin. That is, compromised blood supply in case of ischemic stroke or hemorrhage in case of hemorrhagic stroke. The diminished blood supply in ischemic stroke leads to a decrease in tissue oxygenation, which impairs adenosine triphosphate (ATP) production, leading to dysfunction of ion channels, hence ionic imbalance that causes cellular swelling and tissue edema. In addition, ischemic stress triggers inflammatory cascades, and apoptotic pathways are also activated. Those are thought to be the main mechanisms that contribute to cell injury and death during a stroke event [1]. A subtype of stroke, in which a sudden arterial occlusion or decreased perfusion results in ischemia of brain tissue or the spinal cord, consequent tissue damage, and loss of neurologic function, is known as acute ischemic stroke (AIS), and it is responsible for the vast majority of strokes [2].

Ischemic stroke can occur in the context of atherothrombotic disease, thromboembolism, small vessel disease (lacunar infarct), or other rare conditions, including dissection or vasculitis. In atherothrombotic stroke, damage in the endothelium exposes the subendothelial collagen and the atherosclerotic plaque, which leads to thrombus formation, entrapment of blood cells, and activation of the coagulation cascade, hence rapid occlusion of an artery. In the case of thromboembolic etiology, an embolus originating from proximal vessels or the heart chambers may lodge in a cerebral vessel and cause direct occlusion. On the other hand, small-vessel disease is usually in the background of a lacunar infarct. A thrombotic clot effectively consists of trapped platelets, red blood cells, and a cross-linked fibrin meshwork [3]. Reperfusion therapies aim to dissolve or mechanically remove the clot. Thrombolytic stroke therapy, such as tissue plasminogen activator (tPA), facilitates the cleavage of plasminogen to plasmin, which in turn degrades the fibrin meshwork, leading to clot dissolution [1]. On the other hand, mechanical endovascular thrombectomy (MET) is another reperfusion technique in which an instrument is used to mechanically extract the vessel-occluding thrombus under radiological guidance in case of a large vessel occlusion (LVO) [4].

## Review

Stroke impact and significance

Ischemic stroke is the leading cause of disability worldwide [5]. It is also the leading cause of many morbidities such as dementia and depression. In fact, the second most common cause of dementia is vascular dementia, following Alzheimer’s disease [6]. One in four men and one in five women will develop a stroke if they live up to 85 years, with a recurrence rate of 20-24% after the first stroke episode [7].

A stroke event can lead to life-long disabilities including neurological deficits such as aphasia (inability to speak or communicate), paralysis (inability to move), ataxia, and bowel or bladder dysfunction. The prevalence of stroke varies worldwide, but the absolute number of patients living with stroke-associated disabilities is consistently high. For instance, in Canada, approximately half a million Canadians are living with long-term disabilities due to stroke, and this number is expected to almost double in the next 20 years [4]. In the United States, 800,000 strokes occur per year, which translates to one stroke event every 40 seconds [8].

The estimated direct and indirect cost of stroke care is over $100 billion a year in the United States. Risk factors for stroke are similar to cardiovascular risk factors. They include modifiable risk factors such as hypertension, hyperlipidemia, and diabetes, and lifestyle factors such as smoking, obesity, low physical activity, and an unhealthy diet. While prevention can address those factors, the development of reperfusion therapies and treatment guidelines for effective therapy in the event of stroke is essential to enable stroke survivors to maintain a decent living, with good functional outcomes [6].

What are the core and the penumbra?

**Figure 1:**
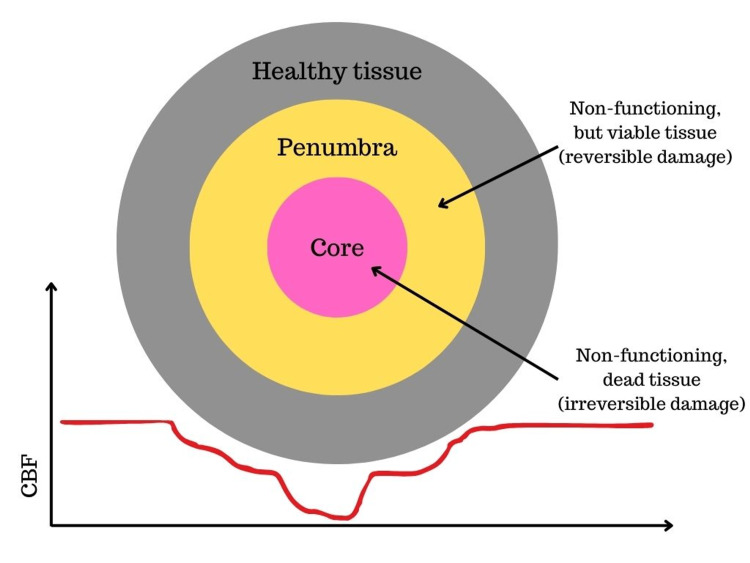
Core and penumbra Illustration of the infarct core (red arrow) and penumbra (green arrow). CBF is plotted. Note the severely reduced CBF in the core region, which results in irreversible tissue damage and death, compared to the less severe reduction of CBF in the penumbral region, which results in reversible damage and preserved tissue viability. This is an original figure produced for the visual illustration of the aforementioned concept. CBF, cerebral blood flow

In the event of a stroke, arterial occlusion results in a rapid decrease in tissue perfusion. In the core of the affected brain area, termed the “infarct core,” blood flow is severely decreased resulting in low ATP levels, disruption in ionic balance, and metabolic failure, which effectively progresses to cell death within minutes [1]. Surrounding the infarct core is an area known as the “ischemic penumbra” or simply the penumbra. Originally, the term was applied to describe brain tissue perfused at levels that are between the functional and morphological thresholds of viability. However, it has been recently extended to refer to viable but hypoperfused brain tissue. Therefore, the infarct core represents an area of dead and irreversibly damaged brain tissue due to severely reduced cerebral blood flow (CBF), while the penumbra represents an area of hypoperfused but viable tissue (Figure 1). While the penumbra is an area of brain tissue, it can also be described as a dynamic process where perfusion and metabolism are impaired in viable neural tissue, which if not rescued can eventually culminate in cell death propagating in a time-dependent manner from the center of ischemia to the surrounding tissue. While the exact mechanisms for the maintenance of the penumbra are not fully understood, it is thought that key processes and molecular mediators are implicated, including but not limited to intermittent waves of depolarization, activation of calcium channels, expression of heat shock proteins, and induction of immediate early genes [9].

During the acute phase of infarct progression, blood flow is below the threshold of basic energy metabolism. This state of energy failure results in an ionic imbalance dictated by the increase in extracellular potassium and intracellular sodium and calcium levels. The elevated extracellular potassium results in waves of depolarization, the so-called peri-infarct depolarization waves, which propagate to the metabolically compromised but still functioning peri-infarct tissue, that is the penumbra. These depolarization waves increase the energy demands of the relevant ion pumps to restore the resting membrane potential. In a normal brain perfusion state, the increase in energy demand is matched with an increase in blood flow. However, in the penumbra, the decreased blood flow prevents this adaptive mechanism. Therefore, a mismatch between the increased metabolic burden and the limited oxygen supply develops, resulting in a stepwise increase in lactate with each depolarization, eventually culminating in cell death [10].

It is noteworthy that the probability of infarction depends not only on the speed of reperfusion therapy administration but also on the severity of the hypoperfusion and the magnitude of collateral supply [11]. In other words, the infarct region expands in a time-dependent manner to occupy the surrounding penumbra, transforming it from viable salvageable tissue to irreversibly damaged tissue. However, a spectrum of fast to slow infarct growth is seen in patients with AIS, potentially reflecting interpersonal differences in patients’ ischemic tolerance and collateral blood supply. That is, in some patients known as “slow progressors,” slow growth of the core into the penumbra is seen, while in other patients known as “fast progressors,” faster propagation of the core is observed. The genetically predisposed availability of leptomeningeal collateral arterioles and their intrinsic hemodynamic features may be in the background of determining whether a patient is a slow or a fast progressor. Local perfusion pressure, arteriolar myogenic reactivity, and early cerebral edema also play a role. Interestingly, those parameters are likely to differ depending on demographics, premorbid conditions, and cerebrovascular risk factors. Thus, further understanding of the determinants of fast and slow progression represents a fresh perspective for understanding stroke pathophysiology [12].

By proposing a viability window rather than a time-window approach, the concept of the penumbra has stirred immense clinical research investigating effective reperfusion therapies that can salvage the viable penumbral tissue in the event of a stroke [9].

The development of stroke therapy

In 1995, the National Institute of Neurological Disorders and Stroke (NINDS) trial marked a new era in stroke therapy when it demonstrated an improved clinical outcome at 90 days in AIS patients who received tPA intravenously, with a number needed to treat (NNT) of 7.7 [13]. In 1996, tPA (i.e., alteplase) was licensed as AIS therapy. However, it was restricted to patients with known symptom onset within a narrow time window of three hours [14]. In 2008, the European Cooperative Acute Stroke Study (ECASS)-3 trial demonstrated the efficacy of intravenous thrombolysis (IVT) with alteplase up to 4.5 hours after symptoms onset [15]. The NNT was 13.7 in this study. In 2014, an individual patient-level meta-analysis of all tPA RCTs further verified the efficacy of tPA in the 0-4.5 hour time window, regardless of age or stroke severity. Nonetheless, given this still narrow time window, only a small proportion of AIS patients were eligible for treatment. Significant differences in thrombolysis rates between regions and countries indicate that patient education, infrastructure, patient transport to the hospital, adherence to guidelines, and establishment of local protocols play a major role in increasing the number of patients suitable for IVT. Moreover, efforts have been focused on improving treatment protocols by identifying eligible patients using tissue viability-based imaging and expediting treatment administration [8].

On the other hand, although the history of endovascular mechanical thrombectomy (MET) is younger than IVT, it has been a rocky road. The motivation for developing MET was the desire to expand the 4.5 hours window of IVT treatment. Three randomized control trials (SYNTHESIS, IMS III, MR RESCUE), alongside multiple single-arm device trials, failed to demonstrate the efficacy of MET. Those failed trials have led researchers to investigate different devices and better patient selection methods. In early 2014, an RCT from the Netherlands (MR CLEAN) demonstrated a positive clinical outcome at 90 days with an NNT of 7.4 in AIS patients with LVO treated with MET within six hours from symptom onset [16]. Five major RCTs followed including Extending the Time for Thrombolysis in Emergency Neurological Deficits (EXTEND) IA, SWIFT PRIME, ESCAPE, REVASCAT, and THRACE, all of which showed positive outcomes. Recently, a meta-analysis of those trials demonstrated an NNT of 5.1 for improved functional outcome at 90 days with MET for patients with LVO stroke if performed within six hours. The success of those recent trials in demonstrating the efficacy of MET was attributed to the strict use of advanced vascular imaging, the use of reliable stenting devices, and the expedited treatment protocols. In 2018, the DWI or CTP Assessment with Clinical Mismatch in the Triage of Wake-Up and Late Presenting Strokes Undergoing Neurointervention with Trevo (DAWN) and Endovascular Therapy Following Imaging Evaluation for Ischemic Stroke (DEFUSE)-3 trials demonstrated the efficacy of MET beyond the six-hour time window in patients presenting with a mismatch in their penumbral imaging studies [8].

Access to and delivery of acute ischemic stroke therapy

A European study across 44 countries has shown that considerable inequalities exist between and within European countries in terms of access to stroke units (SU) and delivery of IVT or MET. That is, only 7.3% of all AIS patients receive IVT, and only 1.9% receive MET. In addition, a significant correlation exists between the number of SU per million inhabitants and the delivery of both IVT and MET. According to the European Brain Council, only one in three acute stroke patients has access to an SU, and at least 628 extra SU would be required across 20 European countries to achieve the benchmark of one SU per one million inhabitants [17].

Those approximate figures are only valid when assuming a uniform distribution of centers across and within countries, which does not usually reflect the real-life situation. For instance, bigger cities and urban areas tend to have more SU, hence their patient population would have better access to acute stroke therapy, compared to remote rural areas. Furthermore, 28 European countries are lacking in Comprehensive Stroke Centers (CSC) where a full range of neurological and neurosurgical services are provided to treat complex stroke patients. That is, at least an additional 286 CSC across 23 countries would be needed to achieve the target standard of care. According to the 2015 Global Burden of Disease Report, the highest country-based IVT rate in Europe was 20.6% (in the Netherlands), while the highest MET rate was 5.6% (in Malta) [17].

The number of people living with stroke in Europe is expected to rise by 27% between 2017 and 2047, owing to population aging and higher survival rates. Variations are expected to continue between nations, indicating that preventive care and stroke management could be improved, particularly in Eastern Europe [18].

Given the above figures, many patients do not have rapid access to SU and, hence, may arrive beyond the approved treatment time window, posing a clinical challenge for the physicians on whether or not to administer IVT or MET. While it is the responsibility of healthcare professionals, stroke specialists, and the relevant authorities to appropriately plan and develop an efficient and affordable network of SU that meets the demands of the patient population, it is important to have treatment options and clinical guidelines in place for patients who arrive to the SU beyond the conventional therapeutic time window [17].

Acute ischemic stroke therapy within the conventional time window

Since 2008, AIS treatment options have been limited to patients with stroke onset within 4.5 hours with IVT being the cornerstone of reperfusion therapy. When a patient arrives at the hospital with acute neurological deficits raising suspicion for stroke or hemorrhage, a contraindication to IVT can be ruled out using a non-contrast CT (NCCT). If no sign of intracranial hemorrhage is seen on the NCCT then the patient likely has an AIS. It is important to establish the time the patient was last seen well, recognize any comorbid conditions, and identify potential contraindications to treatment [19]. If the patient was last seen well within 4.5 hours and no contraindications are present, IVT with tPA, namely alteplase, is initiated. The 2018 Guidelines for Management of Acute Ischemic Stroke recommend that IVT alteplase should be given at a dose of 0.9 mg/kg with a maximum dose of 90 mg over 60 minutes for eligible patients [20].

Following the demonstration of the efficacy of MET for LVO in 2014, it was added to the standard AIS therapy [16]. Hence, in the case of ischemic stroke, CT angiography (CTA) or magnetic resonance angiography (MRA) is also performed to determine whether the stroke is caused by an LVO. This usually follows the NCCT. However, it should never delay the initiation of IVT. In general, the benefit of IVT decreases with time, hence it should be initiated as soon as possible, given that the patient is eligible. Afterward, if the CTA or MRA reveals an LVO, then MET can be performed up to six hours from the symptom onset [21]. Treatment risks should always be weighed against potential benefits, especially in patients presenting with mild stroke [20].

In summary, despite minor variations in treatment protocols between different SU, the standard AIS management generally includes performing an NCCT, establishing the time the patient was last known well, initiating tPA if no contraindications such as intracranial hemorrhage exist and symptoms onset was within 4.5 hours, performing a CTA to identify LVO, possibly followed by MET up to six hours from symptoms onset if an LVO was identified [22]. This time window has been extended further in accordance with specific criteria that will be discussed later.

Recent advances in stroke therapy have allowed the extension of the 4.5 and six-hour time windows of IVT and MET, respectively. This was only possible with the use of advanced functional neuroimaging modalities. However, given the novelty of such guidelines and the sophisticated resources they require, they remain unavailable and yet to be adopted by many SU around the world. Therefore, the standard AIS therapy guideline within the conventional 4.5 to six-hour time window, which has been developed and used over the past two decades, continues to be used to date in many SU [19].

Functional imaging to assess the penumbra and estimate stroke onset

MRI-Based Tissue Clocking: DWI-FLAIR

AIS exhibits a dynamic pattern of development, which can be utilized in neuroimaging to estimate its time of onset. MRI allows for the estimation of stroke onset using the concept of DWI-fluid-attenuated inversion recovery (FLAIR) mismatch, which directly addresses the time window, rather than tissue viability. In other words, it is used to predict the time at which a stroke has occurred. As previously discussed, there is unequivocal evidence for the benefit of IVT with alteplase within the 4.5-hour time window. Therefore, it is invaluable to employ advanced neuroimaging to determine the age of stroke in patients with unknown stroke onset, including wake-up stroke patients [23].

During an ischemic stroke event, the decreased cerebral perfusion leads to a reduction in intracellular energy metabolism, which causes cytotoxic edema, an early marker of ischemic stroke. This edema can be detected with DWI within minutes of stroke onset as an increased signal intensity on DWI and a reduced apparent diffusion coefficient (ADC). In the next hours, the tissue osmolality increases, along with a net increase of water content, which can be detected using T2-weighted MRI three to 4.5 hours after the stroke onset. Therefore, DWI allows for almost instant determination of an AIS lesion but provides no evidence of its further development, which can rather be characterized by T2-weighted MRI. However, an inherent disadvantage of T2-weighted MRI is the high signal intensity from cerebrospinal fluid. Therefore, FLAIR, an MRI sequence that can suppress cerebrospinal fluid effects on the image is preferred [24].

In diagnostic imaging, the pattern of a visible DWI ischemic lesion concurrent with a normal FLAIR, that is, the lack of marked parenchymal hyperintensity on FLAIR (negative FLAIR scan) is termed a DWI-FLAIR mismatch. On the other hand, when the DWI scan detects visible ischemia and the FLAIR scan concurrently shows marked parenchymal hyperintensity, then no mismatch is present. A visual assessment of the DWI and FLAIR scans can identify a mismatch if present. A DWI-FLAIR mismatch was shown to identify patients within three to 4.5 hours of ischemic stroke with high specificity and positive predictive value [25].

A study conducted in 2011 assessed whether a mismatch in DWI-FLAIR imaging can be used to detect patients with unknown time of symptom onset who could be treated with thrombolysis. The study employed 543 patients, and it was found that, indeed, a DWI-FLAIR mismatch identified ischemic stroke lesions with 62% sensitivity, 78% specificity, 83% positive predictive value, and 54% negative predictive value [24].

Furthermore, another study assessed the efficacy of MRI-based thrombolysis for FLAIR-negative stroke patients who presented 4.5 to six hours after stroke onset. DWI-FLAIR mismatch was employed as the selection criterion, and the outcome of thrombolysis was evaluated using the rate of recanalization, the modified Rankin Scale (mRS), and the National Institutes of Health Stroke Scale (NIHSS). The results demonstrated a positive DWI and a negative FLAIR scan, hence a DWI-FLAIR mismatch identified AIS patients with high diagnostic accuracy. The group with stroke onset of zero to 4.5 and the group with stroke onset of 4.5 to six hours who also had a negative FLAIR scan and received IVT had better recanalization rates, mRS, and NIHSS scores than the group with stroke onset of 4.5 to six hours who had a positive FLAIR scan. This further supported the utility of DWI-FLAIR mismatch for identifying patients with stroke onset within 4.5 hours [26].

Penumbral Imaging

Perfusion CT: Perfusion computed tomography (PCT) is an advanced imaging modality used for the evaluation of cerebral perfusion in the setting of cerebral ischemia. It can estimate the volume of the infarct core and the penumbra. Data derived from PCT is presented in perfusion maps as CBF, cerebral blood volume (CBV), mean transit time (MTT), and time-to-maximum (T_max_). The area of the infarct core is the area with severely decreased CBF or CBV, whereas areas with prolonged MTT or its derivatives, the time-to-peak and T_max_ delineate and measure the hypoperfused region including both the core and the penumbra in AIS [27]. CBF and T_max_ are the main parameters used to determine the core and the penumbra in the most widely available software [28].

On Perfusion CT, the most accepted and widely used main parameters to delineate the volume of the core and the penumbra are CBF and T_max_. The core is defined as the area with relative cerebral blood flow (rCBF) less than 30% of the CBF in the contralateral hemisphere. On the other hand, the area with a T_max_ value >6 seconds marks the hypoperfused region, which corresponds to the core and penumbra collectively. The mathematical difference between the volume of the hypoperfused region calculated using T_max_ and the core region calculated using CBF yields the mismatched volume, which corresponds to the volume of the salvageable penumbra. Likewise, the mismatch ratio can be computed as the ratio of hypoperfused tissue (core and penumbra) to the core. The greater the mismatch difference and/or ratio, the larger the penumbra [29].

A retrospective cohort study conducted in 2020 described the prevalence and patterns of abnormal findings in patients who underwent PCT for suspected AIS and were diagnosed with stroke, transient ischemic attack (TIA), or stroke mimic. The PCT imaging abnormalities were compared using IschemaView RAPID software. The results demonstrated that the T_max_ was abnormal (>6 seconds) in 28% of patients with stroke mimic and 62% of patients with stroke/TIA. Hence, patients with stroke mimic were more likely to have a normal T_max_ pattern. In addition, when the T_max_ was abnormal, a greater proportion of patients with stroke mimic had discordant clinical symptoms than patients with stroke/TIA. That is, even though a quarter of the patients with stroke mimics showed T_max_ abnormalities, the majority were discordant with the clinical symptoms [30].

Perfusion CT supports the sensitivity and specificity of the AIS diagnosis, helps in excluding stroke mimics, and informs management and prognosis. However, PCT results in high radiation exposure, increased contrast administration that may culminate in renal injury, increased cost, and increased imaging time that may prolong the time taken to initiate treatment. In addition, PCT techniques vary considerably across institutions depending on the available CT scanners, processing software, and prior functional optimization. Nonetheless, the advantages of PCT seem to outweigh its disadvantages given the potential benefits of increased survival and reduced functional impairment [5].

Perfusion-diffusion MRI: Another penumbral imaging modality that can delineate the core from the penumbra is perfusion-diffusion MRI. Similar to perfusion CT, a magnetic resonance perfusion-weighted image (MR-PWI) delineates the hypoperfused area, which represents the core and the penumbra collectively. T_max_ can be computed from MR-PWI, where a T_max_ value >6 seconds indicates hypoperfusion. On the other hand, MR-DWI is employed to assess the core lesion. It is noteworthy, however, that the DWI abnormality alone does not indicate an ischemic core. Instead, the magnitude of the increase in DWI signal intensity or even more precisely the computed ADC map is suitable for determining the core. The ADC map is calculated from DWI images performed with different b-values (diffusion sensitizing gradient), and an ADC value of ≤620×10^−6^ mm^2^/s or a DWI signal intensity three standard deviations greater than the normal value (SI+3 SD) identifies the ischemic core with a sensitivity of 69% and a specificity of 78% [31].

The mismatch volume can be calculated by subtracting the core volume (obtained using MR-DWI) from the volume of the hypoperfused area (obtained using MR-PWI). The mismatch volume corresponds to the volume of the penumbra. Another measure of the relative volume of the penumbra is the mismatch ratio, which is the ratio of the volume of the hypoperfused area to the area of the ischemic core. The larger the mismatch ratio, the larger the penumbra. In summary, an MR perfusion-diffusion mismatch is an effective diagnostic imaging marker of AIS [32]. However, while MRI can effectively quantify the volume of the penumbra, it remains costly, of long duration, and generally limited in availability worldwide. In addition, a significant number of the AIS patient population cannot be imaged with MRI due to direct contraindications (e.g., pacemaker) or indirect contraindications such as unstable cardiac parameters, agitation and restlessness, aphasia, claustrophobia, or vomiting [23].

MRI-Based Tissue Clocking Versus Penumbral Imaging

Penumbral CT or MRI can identify AIS lesions beyond the conventional 4.5 hour time window. On the other hand, MRI-based tissue clocking can identify AIS that has occurred within 4.5 hours using the concept of DWI-FLAIR mismatch in patients who are unaware of when their symptoms began. The coherence of findings between different studies investigating stroke imaging biomarkers demonstrates how both MRI-based tissue clocking (DWI-FLAIR) and penumbral imaging (MR or CT perfusion imaging) allow for effective selection and identification of AIS patients [25].

Nonetheless, there are pros and cons to both imaging modalities. For instance, DWI-FLAIR mismatch requires MRI but does not require perfusion imaging. It also does not require any post-imaging processing; rather a simple visual assessment suffices. Moreover, a DWI-FLAIR mismatch allows for the treatment of patients with lacunar stroke, which is a subgroup of patients who do not meet the criteria of adequate salvageable tissue in perfusion-based penumbral mismatch imaging techniques. On the other hand, penumbral imaging might identify eligible patients with salvageable tissue despite the presence of hyperintensity on FLAIR. Therefore, penumbral imaging can detect stroke with a significant size of penumbra beyond the 4.5 hour time window [33].

In summary, the key difference between the concept of DWI-FLAIR mismatch and perfusion-diffusion imaging is that DWI-FLAIR imaging reveals information about the time of stroke onset, while penumbral imaging assesses the extent of tissue damage and viability [25].

In conclusion, DWI-FLAIR mismatch aims to identify stroke that has occurred within 4.5 hours in patients who are unaware of when their symptoms began or have awakened with stroke symptoms. On the other hand, penumbral imaging can identify stroke patients with a significant size of penumbra in whom the time of symptom onset is known to be more than 4.5 to six hours or in whom the time of symptom onset is unknown [33].

Impact of functional imaging on reperfusion therapies

Imaging-Based Intravenous Thrombolysis

IVT for stroke with a known late time window: The introduction of functional neuroimaging including penumbral imaging into stroke management has allowed for the identification of AIS patients who are still eligible for IVT due to large penumbra despite presenting late to the SU. Several trials have been conducted to investigate IVT eligibility in patients who present with stroke symptoms beyond the conventional 4.5-hour time window, the most remarkable of which are the ECASS-4, EXTEND, and EPITHET trials [34].

A guideline was developed by the European Stroke Organization (ESO) to assist physicians in determining the eligibility of AIS patients with late presentation for IVT therapy. The guideline was developed based on scientific evidence that surpasses the quality of evidence published in other stroke recommendations and earlier guidelines. It was mostly based on RCTs and individual-participant data meta-analyses rather than observational studies. However, whenever insufficient evidence was available to make an evidence-based recommendation, an experts’ consensus statement was provided [19].

In this guideline, the question of whether IVT with alteplase would lead to a better functional outcome in patients with a known AIS onset of 4.5 to nine-hour duration who also demonstrate a core-perfusion mismatch with penumbral imaging including perfusion CT or MRI was proposed. However, it was found that most RCTs of extended time windows included not only patients with a known stroke onset of 4.5 to nine hours but also wake-up stroke patients. A wake-up stroke is a situation in which a patient awakens with stroke symptoms that were not present prior to falling asleep. Patients who cannot report the onset of stroke but were symptom-free when they were last seen belong also to this subgroup. Despite being handled similarly in most RCTs, those two types of strokes (known stroke onset of 4.5 to nine hours and wake-up stroke) represent two different clinical scenarios since the true onset of stroke in wake-up stroke patients was shown to frequently occur in the early morning hours, often less than 4.5 hours prior to awakening. Considering the original aim of targeting patients with a known onset of 4.5 to nine hours, those two groups were handled separately, and distinct recommendations were developed for each [19].

The ECASS-4 investigated the benefit of IVT alteplase compared to placebo in patients with stroke onset between 4.5 and 9 hours. MRI core-perfusion mismatch was used to select patients for treatment. An imaging criterion was developed for the inclusion of patients. The criteria were the demonstration of an infarct core volume of <100 mL, an absolute hypoperfusion lesion volume of at least 20 mL, and a >1.2 mismatch ratio between the hypoperfusion lesion and the core. The concern with this study was that 69% of the patients were wake-up stroke patients, hence potentially with a stroke onset of less than 4.5 hours as explained above, whereas only the remaining 31% had a known stroke onset of 4.5 to nine hours. Nonetheless, there was no significantly better functional outcome at three months between the two subgroups compared to placebo demonstrating an OR of 1.20 (95% CI: 0.63-2.27, P=0.57), where OR is the odds ratio, and 95% CI is 95% confidence interval range [35].

Moreover, the EXTEND trial had an adjusted inclusion criterion of patients where only patients with an infarct volume of <70 mL, an absolute hypoperfusion lesion of >10 mL, and a >1.2 mismatch ratio between the hypoperfusion lesion and the core were deemed eligible. Both MRI DWI-PWI and CTP penumbral imaging modalities were allowed in the study. Similar to the ECASS-4 study, only 35% of the recruited patients had a known stroke onset of 4.5 to nine hours, while the rest had woken up with a stroke. The median time between the onset of stroke and the initiation of treatment was 7.5 hours. The EXTEND trial demonstrated that the percentage of patients who received IVT therapy with excellent outcomes at three months of follow-up was higher (35.4% versus 29.5%) and there was no difference between patients treated during different time sub-intervals (4.5 to six hours or six to nine hours). The null hypothesis was, therefore, rejected when an adjusted RR of 1.44 (95% CI (1.01-2.06)), where RR is the relative risk (also known as the risk ratio) and 95% CI is the 95% confidence interval range, was found between the proportion of patients with excellent functional outcome (mRS score: 0-1) at three months and neuroimaging evidence of recanalization in 24 hours in the intervention group compared to the control group. However, the risk of symptomatic intracerebral hemorrhage (sICH) was notably higher in the IVT alteplase-treated group (adjusted RR: 7.22, 95% CI (0.97-53.5)) [36].

Furthermore, in a pooled analysis of the EPITHET trial and another observational study, it was found that 60% of patients with known stroke onset of three to six hours who had an MR DWI-PWI mismatch and were treated with IVT alteplase had a significantly smaller infarct size. However, their clinical outcome was similar to the placebo group [37].

A systematic review of the EXTEND, ECASS-4, and EPITHET trials and other relevant studies conducted between 2006 and 2019 aimed to determine whether penumbral imaging can help identify AIS patients with salvageable brain tissue who presented between 4.5 to nine hours after the onset of stroke symptoms or who woke up with stroke symptoms. It was found that the number of patients treated with alteplase who achieved excellent functional outcomes at three months (mRS score: 0-1) was greater than the placebo group (adjusted OR: 1.86, 95% CI (1.15-2.99)). However, sICH was more prevalent in the alteplase group than the placebo group (adjusted OR: 9.7, 95% CI (1.23-76.55)). There was no statistically significant difference between mortality at three months between the two groups (adjusted OR: 1.55, 95% CI (0.81-2.96)) [38].

However, because 51% of the patients in this review experienced a wake-up stroke, a stratified analysis was done for only the patients who had a known stroke onset of 4.5 to nine hours. Nonetheless, a similar result was demonstrated. A greater proportion of patients achieved excellent functional outcome (mRS score: 0-1) (OR: 2.23, 95% CI (1.10 - 4.50)) or good functional outcome (mRS score: 0-2) (OR 2.14, 95% CI (1.07 - 4.28)) at three months in the alteplase-treated group compared to the placebo group [38].

In the above-discussed studies, it is notable that 62% of the patients analyzed did not undergo MET. Therefore, the ESO strongly recommends IVT with alteplase for patients with a known stroke onset of 4.5 to hours hours given that CT or MRI core-perfusion mismatch was demonstrated and for whom MET is either not indicated or not planned. However, there is no current RCT-based evidence to decide whether IVT is of any benefit in patients with no core-perfusion mismatch. Therefore, for patients with a stroke onset of 4.5 to nine hours but no CT or MRI core-perfusion mismatch, all group members of the ESO guideline recommend against treatment with IVT with alteplase. Finally, there is currently insufficient data to make an IVT recommendation for patients who are scheduled to undergo MET and who are also eligible for IVT in the 4.5 to nine-hour time window based on the imaging inclusion criteria [19].

IVT for stroke with an unknown time window: As previously mentioned, IVT with tPA (i.e., alteplase) was only approved for patients with AIS presenting within 4.5 hours (three hours in the US, Canada, Croatia, and Moldova). This continued to be followed until the WAKE-UP trial, a multicenter, randomized, double-blinded, placebo-controlled trial was published [39].

The WAKE-UP trial used DWI-FLAIR mismatch for the selection of patients eligible for IVT beyond the 4.5 hour time window based on a certain inclusion criterion. The question was whether IVT with alteplase improves clinical outcomes in AIS patients presenting with an unknown stroke onset, given that their MRI studies demonstrate a DWI-FLAIR mismatch. Out of the 503 enrolled patients in eight European countries, 254 were assigned to receive alteplase and 249 to receive placebo. The median NIHSS score at the time of initial examination was six in both groups. The most common reason for the unknown time of stroke onset was that the patient had awakened from nighttime sleep with stroke symptoms (89% of patients in both groups). It was found that a favorable functional outcome at 90 days, which is the primary endpoint, was higher among the patients who received IVT with alteplase than the patients who received placebo (adjusted OR: 1.61, 95% CI (1.09-2.36)) [39].

Another trial that utilized CT or MRI core-perfusion imaging for wake-up stroke patients was the EXTEND trial [36]. The study recruited patients based on CT or MRI core-perfusion mismatch and compared an IVT patient group to a placebo control. As discussed earlier, the EXTEND trial included both patients with known stroke onset of 4.5 to nine (79 of 225 patients, 35%) hours and patients with an unknown symptom onset including wake-up stroke patients (146 of 225 patients, 65%) [36].

Evaluation of unknown Onset Stroke thrombolysis trials (EOS) investigators performed a systematic review and individual patient data meta-analysis of those RCTs of IVT with alteplase in which patients with unknown stroke onset were included and advanced imaging (DWI-FLAIR mismatch or penumbral imaging) were used for patient selection. As these trials used different imaging techniques (DWI-FLAIR mismatch in the WAKE-UP and THWAS trials, and core-perfusion mismatch on penumbral imaging in the EXTEND and ECASS-4 trials), and some of these trials included patients not only with unknown but also with known stroke onset; the EOS investigators re-analyzed the data of those patients who presented with unknown stroke onset. In the re-analysis, the definition of penumbral mismatch used in the EXTEND trial was applied (infarct core volume of <70 mL, absolute perfusion lesion volume of >10 mL, and a >1.2 mismatch ratio between hypoperfused area and core). It should also be mentioned that contrary to the other trials, the stroke onset in the EXTEND trial was calculated from the midpoint of sleep and not from the last seen well time-point. Re-analysis of 843 patients demonstrated that IVT with alteplase was associated with an excellent outcome (adjusted OR: 1.49, 95% CI (1.10-2.03]) and better functional outcome (adjusted OR: 1.39, 95% CI (1.05-1.80)), while it was associated with a higher risk of sICH (3% vs. 0.5%, p=0.02) and mortality within three months (adjusted OR: 2.06, 95% CI (1.03-4.09)) [33].

The strength of evidence based on which the recommendations were made was rated as high due to the relatively large sample size of patients and the consistency of results observed. Given the above, IVT with alteplase is recommended for patients with AIS on awakening from sleep who were last seen well more than 4.5 hours ago and with an MRI DWI-FLAIR mismatch given that MET is not indicated or not planned. Similarly, IVT with alteplase is strongly recommended for patients with AIS on awakening from sleep who show a CT or MRI core-perfusion mismatch within nine hours from the midpoint of sleep given that MET is not indicated or not planned. It is notable, however, that the midpoint of sleep, which was the parameter used only in the EXTEND trial, was employed in this meta-analysis for patients with stroke of unknown onset, despite employing the “last seen well” time parameter in the rest of the included trials. On the other hand, for patients presenting with wake-up stroke and LVO who are eligible for both IVT and MET, an expert consensus statement was made in favor of IVT before the MET [19].

Imaging-Based Mechanical Endovascular Thrombectomy

MET based on penumbral imaging: Another routinely used effective stroke therapy is MET, a form of endovascular treatment. Endovascular therapy refers to the removal of a clot aiming to restore blood flow using mechanical devices, hence often referred to as endovascular or MET. It is an effective stroke therapy, with a recanalization success rate of 88% if appropriate FDA-cleared thrombectomy devices (stent-retrievers) or suction thrombectomy systems are used. Until recent years, there was no randomized data available regarding the safety and efficacy of endovascular therapy (i.e., MET beyond six hours from symptom onset) [40].

The DEFUSE-3 trial was conducted to investigate the efficacy of endovascular therapy in reducing the degree of disability three months after the stroke episode in patients with LVO who present beyond six hours from symptom onset given they have a favorable neuroimaging profile. The goal of the study was to shift the selection of patients for late reperfusion therapy to an objective clinical decision based on scientific evidence. The study included patients treated between six and 16 hours of the symptom onset and used an automated software (RAPID) to determine if a mismatch is present in CT perfusion or MR diffusion-perfusion. The imaging criteria used in the DEFUSE-3 trial had an infarct core volume of <70 mL, a mismatch volume of ≥15 mL, and a mismatch ratio of ≥1.8 in a patient who is at least 18 and at most 90 years old and an NIHSS score of at least six points. The comparison groups included the test group who received endovascular therapy plus medical therapy and a control group who received only medical therapy. The results demonstrated that patients with an LVO and a core-perfusion mismatch who presented after six hours of symptoms onset and received endovascular therapy within 16 hours achieved better clinical outcomes and less infarct growth, as opposed to patients who only received medical therapy (adjusted OR: 3.36, 95% CI (1.96-5.77)). A higher percentage of patients showed good clinical outcomes (mRS score: 0-2) at 90 days in the thrombectomy group (45%) than in the control group (17%; p<0.001). Moreover, the 90-day mortality rate was lower if endovascular therapy was performed (14%) compared to those patients who received only medical therapy (26%; p=0.05) [41].

In addition to providing scientific evidence for the efficacy of endovascular therapy in the treatment of AIS beyond the six-hour time window, the DEFUSE-3 trial also provided data that allowed for the correlation between angiographic findings before and after endovascular therapy with baseline and follow-up imaging findings. A study utilized these data from the DEFUSE trial to review the correlation between the angiographic findings and the clinical outcome, mechanical device choice, and noninvasive imaging outcomes. Angiograms were assessed using the modified thrombolysis in cerebral infarction (TICI) score at baseline and after treatment, whereas the clinical outcomes were assessed using the mRS. Then, the correlation between the TICI scores and the clinical outcome, the device used, and the 24-hour follow-up imaging results were examined. A TICI score of 2B or 3 represented 50-99% or 100% (complete) reperfusion, respectively. Hence, a TICI score of 2B-3 was considered a benchmark for successful reperfusion. With a better TICI score, the number of patients with good functional outcomes (90-day mRS score of 0-2) showed a statistically significant increase (OR: 2.77, 95% CI (1.17 - 6.56)). Moreover, a decrease in infarct growth at 24 hours was noted in the TICI 3 patients, compared to the TICI 0-2B patients. Furthermore, successful reperfusion (TICI 2B-3) had no correlation with the MET device used, the site of occlusion, or the adjuvant use of carotid angioplasty or stenting. The reperfusion success rate of 76% with endovascular therapy in the extended time window (six to 16 hours) seen in the DEFUSE 3 trial was also consistent with the rates achieved in earlier time window trials such as the HERMES trial (70%) and the recently reported DAWN trial (84%) [42].

MET based on infarct volume and clinical deficit: The other clinically significant trial was the DAWN trial. It assessed the potential benefit of MET in an extended time window of six to 24 hours in patients with a disproportionately more severe clinical deficit, compared to the infarct volume. The patient selection criteria took into account the patient's age, the infarct volume, and the clinical deficit assessed using the NIHSS score. The coprimary endpoints of the study were the mRS at 90 days and the mean score for disability on the utility-weighted mRS (which ranges from 0 (death) to 10 (no symptoms or disability)). The study included 206 patients who were divided into a thrombectomy group and a control group, and the results demonstrated better outcomes in the treatment group in terms of the mRS at 90 days, which was 5.5 and 3.4 in the thrombectomy and control group, respectively. Overall, the rate of functional independence at 90 days defined as an mRS score of 0-2 was also higher in the thrombectomy group (49% and 13% in the thrombectomy and control groups, respectively). In addition, the rate of stroke-related death and death from any cause at 90 days did not show a statistically significant difference between the treatment and the control groups (RR: 1, CI (1-2)). Therefore, AIS patients who present six to 24 hours after symptom onset with a mismatch between clinical deficit and infarct volume achieve better outcomes when treated with endovascular thrombectomy in addition to standard care, as opposed to being treated with standard care alone [43].

MET for AIS with a large infarct: Recent clinical trials published in 2022 and 2023 investigated the safety and efficacy of MET performed within 24 hours of stroke onset for AIS with a large ischemic core, based on dynamic neuroimaging studies. Because MET is usually avoided when the infarction is large, the effect of MET with medical treatment as opposed to medical treatment alone has not been well-studied. A clinical trial in Japan involving 203 patients with AIS and a large infarct demonstrated that those who received MET in addition to medical treatment had better functional outcomes assessed using the mRS score, than those who received the latter alone. However, they also had a higher incidence of subsequent intracranial hemorrhages [44]. Similarly, another trial of a limited population of 352 patients demonstrated better functional outcomes in the MET group but also a higher incidence of vascular complications such as access-site complications, dissection, cerebral vessel perforation, and transient vasospasm [45]. Finally, a randomized clinical trial in China involving 456 LVO AIS patients with a large infarct revealed a similar conclusion for the efficacy of MET with medical therapy versus medical therapy alone [46].

Changes in acute ischemic stroke management

Modified stroke therapy guidelines currently aim to provide rapid reperfusion therapy with IVT or MET, both of which have demonstrated efficacy in reducing treatment disability in an extended time window. That is, while IVT reduces disability when given to AIS patients within 4.5 hours of stroke onset, the time window could be extended from 4.5 to nine hours in selected patients with evidence of salvageable brain tissue based on penumbral imaging. On the other hand, while MET reduces disability when administered within six hours in AIS patients with evidence of LVO, the time window could be extended up to 24 hours following stroke onset in selected patients with a core-perfusion mismatch on penumbral imaging. In the following paragraphs, the updated recommendations for IVT and MET based on functional neuroimaging are summarized [20].

Regarding the eligibility for IVT, if a patient presents with symptoms of AIS within a known time window of 4.5 to nine hours, an MR diffusion and perfusion or CT perfusion scan should be performed. If a core-perfusion mismatch is found and MET is not indicated or planned, then IVT with alteplase is recommended. If a core-perfusion mismatch was not found, then IVT is not recommended. On the other hand, if a core-perfusion mismatch is found and the patient is eligible for MET and MET is not directly available, then IVT can be given as bridging therapy until MET can be performed. Finally, if a patient presents with a core-perfusion mismatch and is eligible for MET, which is readily available (e.g., due to presenting directly to a thrombectomy center), then no recommendation could be made for the administration of IVT before MET due to insufficient evidence [19].

If, however, the patient presents with an unknown time window due to awakening from sleep with stroke symptoms or any other reason (e.g., aphasia), then either MRI-based tissue clocking or penumbral imaging could be performed. If MRI demonstrates a DWI-FLAIR mismatch in a patient last seen well >4.5 hours ago and MET is not indicated or planned, then IVT with alteplase is strongly recommended. Similarly, if an MR DWI-PWI or CT perfusion scans demonstrate a core-perfusion mismatch in a patient presenting within nine hours from the midpoint of sleep for wake-up stroke or from the unknown time period, and MET is not indicated or planned, then IVT is also recommended. Finally, if either imaging modality demonstrates a core-perfusion mismatch in a patient presenting with unknown symptom onset, and MET is indicated but not directly available, then IVT can be administered as bridging therapy until MET can be performed [19]. Moreover, in the case of core-perfusion mismatch in a patient with unknown symptom onset, and the patient is eligible for both IVT and MET, and MET is directly available, then no recommendation could be made for the administration of IVT before MET due to insufficient evidence [19].

Regarding the eligibility for MET, if a patient presents at least six hours after symptom onset and CTA demonstrates an LVO, then either an MR diffusion and perfusion or a CT perfusion scan can be performed. If a core-perfusion mismatch is demonstrated and the patient meets the DEFUSE-3 or DAWN MET criteria, then MET is recommended up to 16 or 24 hours from symptom onset, respectively [20].

Limitations

While various studies have demonstrated the efficacy of IVT beyond the 4.5 hour time window, there remain limitations to both the study designs and their applicability in clinical practice. For instance, the introduction of another calculation method for assessing the duration of stroke in patients with unknown symptom onset made it difficult to draw appropriate conclusions from different trials [19].

Besides, wide variations in imaging protocols, inconsistent post-processing, and relatively small sample sizes are likely to hinder the successful efforts to utilize functional imaging biomarkers for thrombolysis beyond the 4.5-hour time window based on CTP or MRI perfusion-diffusion mismatch patterns. In addition, the advances in stroke therapy come at the expense of increased complexity, which will likely require alterations in the existing treatment protocols. Moreover, imaging devices and analysis software are expensive resources that may not always be available in smaller stroke care facilities [47]. 

With respect to this review, a possible limitation is the inclusion of trials and studies that only investigated the use of alteplase for IVT, without parallelly including ones that utilized other thrombolytic agents, such as tenecteplase. Despite its comparable safety and efficacy profile, tenecteplase use has increased since the COVID-19 pandemic due to its shorter preparation and administration time as a means of reducing exposure to the COVID-19 virus during stroke emergencies. Therefore, a review of its utility in the context of the extended treatment window would be relevant [48].

## Conclusions

Over the past few decades, stroke therapy has improved significantly, going from therapeutic nihilism to offering multiple proven treatment options. Intravenous thrombolysis and MET are the two main modalities of reperfusion therapy that used to be limited to patients presenting within a narrow time window of 4.5 and six hours, respectively. However, with advances in neuroimaging modalities such as MRI tissue-based clocking and penumbral imaging paralleled with relevant randomized clinical trials, the time window has been extended to nine hours for intravenous thrombolysis and 16 or 24 hours for MET, depending on whether the DEFUSE-3 or DAWN criteria is utilized for patient eligibility.

With the ability to utilize neuroimaging to select reperfusion therapy candidates, we would be able to improve functional outcomes for a large population of AIS patients who present late or with an unknown time of stroke onset, often due to awakening with stroke symptoms. In other words, multimodal imaging could potentially transform stroke therapy from a time window-based to a tissue viability-based approach. While these advances aim to extend the time window and improve treatment options for late arrivals and for strokes of unknown time onset, it must be recognized that trials are ongoing, and more research is recommended to elaborate on treatment options. Overall, stroke remains an emergency, and time is still brain. Therefore, emphasis should be placed on expediting treatment, while utilizing the growing imaging complexities that could guide advanced clinical decision-making.
